# Comparison of Doxycycline, Minocycline, Doxycycline plus Albendazole and Albendazole Alone in Their Efficacy against Onchocerciasis in a Randomized, Open-Label, Pilot Trial

**DOI:** 10.1371/journal.pntd.0005156

**Published:** 2017-01-05

**Authors:** Ute Klarmann-Schulz, Sabine Specht, Alexander Yaw Debrah, Linda Batsa, Nana Kwame Ayisi-Boateng, Jubin Osei-Mensah, Yusif Mubarik, Peter Konadu, Arcangelo Ricchiuto, Rolf Fimmers, Sandra Arriens, Bettina Dubben, Louise Ford, Mark Taylor, Achim Hoerauf

**Affiliations:** 1 Institute for Medical Microbiology, Immunology and Parasitology, University Hospital Bonn, Bonn, Germany; 2 Institute for Medical Biometry, Informatics and Epidemiology, University Hospital Bonn, Bonn, Germany; 3 German Center for Infection Research (DZIF), partner-site Bonn-Cologne, Bonn, Germany; 4 Institute of Laboratory Animal Science, University of Zurich, Zurich, Switzerland; 5 Faculty of Allied Health Sciences, Kwame Nkrumah University of Science and Technology, Kumasi, Ghana; 6 Kumasi Centre for Collaborative Research in Tropical Medicine (KCCR), Kumasi, Ghana; 7 University Hospital, Kwame Nkrumah University of Science and Technology, Kumasi, Ghana; 8 School of Medical Sciences, Kwame Nkrumah University of Science and Technology, Kumasi, Ghana; 9 Department of Parasitology, Liverpool School of Tropical Medicine, Liverpool, United Kingdom; Imperial College London, Faculty of Medicine, School of Public Health, UNITED KINGDOM

## Abstract

The search for new macrofilaricidal drugs against onchocerciasis that can be administered in shorter regimens than required for doxycycline (DOX, 200mg/d given for 4–6 weeks), identified minocycline (MIN) with superior efficacy to DOX. Further reduction in the treatment regimen may be achieved with co-administration with standard anti-filarial drugs. Therefore a randomized, open-label, pilot trial was carried out in an area in Ghana endemic for onchocerciasis, comprising 5 different regimens: the standard regimen DOX 200mg/d for 4 weeks (DOX 4w, N = 33), the experimental regimens MIN 200mg/d for 3 weeks (MIN 3w; N = 30), DOX 200mg/d for 3 weeks plus albendazole (ALB) 800mg/d for 3 days (DOX 3w + ALB 3d, N = 32), DOX 200mg/d for 3 weeks (DOX 3w, N = 31) and ALB 800mg for 3 days (ALB 3d, N = 30). Out of 158 randomized participants, 116 (74.4%) were present for the follow-up at 6 months of whom 99 participants (63.5%) followed the treatment per protocol and underwent surgery. Histological analysis of the adult worms in the extirpated nodules revealed absence of *Wolbachia* in 98.8% (DOX 4w), 81.4% (DOX 3w + ALB 3d), 72.7% (MIN 3w), 64.1% (DOX 3w) and 35.2% (ALB 3d) of the female worms. All 4 treatment regimens showed superiority to ALB 3d (*p* < 0.001, *p* < 0.001, *p* = 0.002, *p* = 0.008, respectively), which was confirmed by real-time PCR. Additionally, DOX 4w showed superiority to all other treatment arms. Furthermore DOX 4w and DOX 3w + ALB 3d showed a higher amount of female worms with degenerated embryogenesis compared to ALB 3d (*p* = 0.028, *p* = 0.042, respectively). These results confirm earlier studies that DOX 4w is sufficient for *Wolbachia* depletion and the desired parasitological effects. The data further suggest that there is an additive effect of ALB (3 days) on top of that of DOX alone, and that MIN shows a trend for stronger potency than DOX. These latter two results are preliminary and need confirmation in a fully randomized controlled phase 2 trial.

**Trial Registration:** ClinicalTrials.gov #06010453

## Author Summary

Onchocerciasis is a vector borne disease that is still a major health burden in endemic countries, despite twenty years of control that has led to effective control and reduction of blindness and morbidity in West Africa. To reach the goal of onchocerciasis elimination, alternative strategies are needed to overcome existing hurdles. Ivermectin is used for mass drug administration (MDA) and kills the microfilariae, thus preventing uptake by the vector and reducing transmission. Since adult worms live for 10 years or longer, MDA requires many years of treatment, a heavy burden on health care systems. Inadequate population coverage, sub-optimal responses, possible development of resistance and the risk of severe adverse reactions with co-endemic Loiasis are current hurdles for achieving elimination. The search for new drugs that could enhance elimination by permanently sterilizing and killing the adult worms has identified a 4–6 weeks course of doxycycline, a slow-killing drug due to its indirect mode of action by killing endosymbiotic bacteria. The present study aimed to investigate a new anti-*Wolbachia* drug, minocycline, and doxycycline in combination with one of the standard broad-spectrum anti-helminthic drugs, albendazole, to identify shorter treatment regimens. Our data confirm the efficacy of a 4-weeks doxycycline regimen and show that albendazole has additive effects when used in combination with doxycycline. In addition, comparison of a 3-week course of minocycline with a 3-week course of doxycycline revealed a trend for stronger efficacy of minocycline. Further development of anti-*Wolbachia* and anti-filarial drug combinations are warranted to improve treatment options to overcome existing hurdles and for use in “end-game” scenarios when switching from MDA to “test & treat” strategies.

## Introduction

More than 200 million humans are parasitized by filarial nematodes, causing the neglected tropical diseases: lymphatic filariasis, loiasis and onchocerciasis. The lymphatic, ocular and dermatological pathologies have severe economic and social consequences including poor school performance, low productivity, higher health related costs among infected adults and a reduced life span [1–3]. In onchocerciasis, several programs and developments have greatly improved the situation in Africa since the 1970’s when the Onchocerciasis Control Programme (OCP), a programme relying on vector control, in West Africa was initiated. In 1987, a new treatment-based strategy was made possible due to the donation of ivermectin (IVM) by Merck as long as needed and the African Programme for Onchocerciasis Control (APOC), a coordinated community directed distribution of IVM mass drug administration (MDA) in 28 African countries [4] that has now ended in 2015, was launched. Despite the tremendous progress [5], disease elimination will require continuity and new approaches with APOC and onchocerciasis control are being integrated into the Expanded Special Project for Elimination of Neglected Tropical Diseases (ESPEN).

A major problem with the current treatment is that IVM has minimal efficacy against adult worms and does not permanently stop microfilariae (Mf) production. Simulation studies have suggested that administering IVM at shorter intervals of 6 instead of 12 months intervals have a higher likelihood of eliminating the infection, but incur large logistical costs on health infrastructures of the endemic countries [6, 7]. Immigration of infected persons into areas where filariasis is considered eliminated and IVM treatment has ceased may occur with potential re-emergence of the disease. Equally important is the evidence of persistent IVM suboptimal efficacy in some communities [8–11], which is of particular concern as there is currently no alternative treatment suitable for MDA.

In addition, MDA cannot easily be undertaken in regions endemic for onchocerciasis, where patients are co-infected with *Loa loa* (primarily in Central Africa) and which are estimated to cover at least 20% of onchocerciasis-endemic areas [12]. This is because microfilaricidal drugs also kill *Loa loa* Mf and in patients with high parasitemia this may elicit loiasis-specific adverse reactions (*Loa loa* encephalopathy), leading to severe neurological disorders or death [13]. Thus there is an urgent need for a safe macrofilaricide, targeting adult worms that can be used for onchocerciasis and LF, particularly in problem areas, such as those with emerging ivermectin resistance or *Loa loa* co-endemicity. Further development of anti-*Wolbachia* macrofilaricides is therefore warranted to improve treatment options to overcome existing hurdles and for use in “end-game” scenarios in areas with hypoendemicity or when switching from MDA to “test & treat” strategies (who.int/apoc/ATS_Report_2015.12).

Targeting *Wolbachia* endosymbionts by antibiotics is an alternative approach that has been verified by a number of clinical trials in onchocerciasis [14–16] and LF [17–21]. Depletion of *Wolbachia* leads to long-term sterilization and to a macrofilaricidal effect. This has been shown first in animal models [22–24] and was successfully translated into clinical trials in human onchocerciasis [15] and lymphatic filariasis [21, 25]. A recent meta-analysis on the available data from clinical trials in onchocerciasis has shown that the 3 so far used regimens, doxycycline (DOX) 200 mg/day for 4 weeks, DOX 200 mg/day for 6 weeks [15], and DOX 100 mg/day for 5 weeks [26] are broadly equivalent, in particular with regards to the sterilizing effects of adult female worms [27], and therefore DOX treatment with 200 mg/day for 4 weeks can be considered as a “standard” anti-wolbachial therapy for onchocerciasis.

The advantages of DOX for the depletion of *Wolbachia* endobacteria have stimulated the development of new drugs. The indirect mode of action with its slow antifilarial activity confers an excellent safety profile by avoiding inflammatory reactions [28] and can safely be used in *L*. *loa* coendemic areas, since this filarial parasite does not contain *Wolbachia*. The A∙WOL Consortium was established to find new anti-wolbachial drugs or drug combinations that are deliverable in a shorter regimen, with a secondary goal to optimize regimens using currently known anti-*Wolbachia* antibiotics (www.a-wol.com). The tetracycline derivate minocycline (MIN), was identified as priority hit in the cell culture screening assay [29] as well as on adult *O*. *gutturosa* male worms [3] and in the *L*. *sigmodontis* animal model [30]. Albendazole (ALB) is widely used in combination with IVM for lymphatic filariasis and soil transmitted helminths, yet no clear evidence for efficacy against onchocerciasis has been provided.

Here, we present the results of a randomized open pilot-trial to compare DOX, MIN, as well as the combination of DOX plus ALB to ALB alone in their efficacy against onchocerciasis. The underlying rationale that led to the design of this trial was to provide a shortened treatment that is equally efficient in *Wolbachia* depletion or blockage of embryogenesis after 6 months as the current regimen of 4 weeks DOX 200mg compared to 3 days ALB 800 mg.

## Materials and Methods

### Study population and ethics statement

This study was undertaken in 14 villages adjacent to the river Offin in Ghana (Upper and Lower Denkyira Districts in the Central Region and the Amansie Central and Adanse South Districts in the Ashanti Region). These rain forest areas are within the distribution range of the vector (<12 km from the breeding site, the river Offin and affluent creeks) and hyperendemic for onchocerciasis but not for other filarial infections and were not part of either OCP or APOC programs. MDA has been implemented by the Ghanaian Ministry of Health since 2001 in Upper and Lower Denkyira districts and from 2008 in the Amansie and Adanse South areas. However, MDA compliance has not met MDA targets in all areas, especially in the Upper and Lower Denkyira districts, and therefore, at the time of sampling a considerable number of people had not or not frequently taken part in IVM therapy. This study was approved by the Committee on Human Research, Publications and Ethics of the School of Medical Sciences of the Kwame Nkrumah University of Science and Technology (KNUST), Kumasi, Ghana,—Research Ethics Committee of the Liverpool School of Tropical Medicine as well as the Ethical Committee of the University Hospital of Bonn, Germany. The trial was registered at Current Controlled Trials (www.isrctn.com, # 06010453).

Eligible for this study were healthy male and female patients (without clinical conditions requiring long-term medication and normal renal and hepatic laboratory profiles), aged between 18–55 years, > 40 kg of body weight and the presence of at least one palpable onchocercoma. Hepatic and renal functions as well as pregnancy tests were assessed by dipstick chemistry using venous blood and urine. Skin snips (biopsies) were taken to determine skin microfilarial (Mf) loads. Exclusion criteria were: abnormal hepatic and renal enzymes, pregnancy, breast-feeding, intolerance to the study drugs, alcohol or drug abuse, history of TB, glucose in urine, hypertension or history of any condition requiring long term medication (see also ISRCTN registry). Written informed consent was obtained from all individuals. A Data Monitoring and Ethic Committee (DMEC) was established prior to this study.

### Study design, randomization and interventions

This study is a randomized, open, clinical phase 2a drug trial. The purpose of the 5 different treatment regimens was to determine if a reduced treatment period could be achieved using doxycycline 200mg alone for 3 weeks (DOX 3w), doxycycline 200mg for 3 weeks plus 3 days albendazole (DOX 3w+ALB 3d), minocycline 200mg for 3 weeks (MIN 3w) or doxycycline 200mg for 4 weeks (DOX 4w) as standard anti-wolbachial regimen compared to albendazole 800mg for 3 days (ALB 3d), which is considered equivalent to an untreated control arm, when given on its own [31].

This study was a pilot trial to gain first experience regarding the primary and secondary endpoints under the intended treatment regimens. Therefore a sample size of 30 participants (20 participants plus 50% drop-out rate) per treatment arm was chosen in line with the sample sizes normally taken for pilot trials. Participants were randomly assigned to one of 5 treatment arms according to their participation in the MDA and their Mf-status as follows:

1. Mf-positive (> 10 Mf/mg skin) or > 2 palpable onchocercomata: no limitation of ivermectin (MDA) rounds;
2. Mf-positive (0.1–10 Mf/mg skin) and ≤ 2 palpable onchocercomata: last ivermectin treatment > 1 year ago and not more than 3 rounds;
3. Mf-negative (0 Mf/mg skin) ≤ 2 palpable onchocercomata: last ivermectin treatment > 1 year ago and not more than 1 round.

Participants received the study drugs under daily observation. Participants were given IVM after the nodulectomies. After study completion, DOX 4w was offered to all participants. Even though the study was an open trial and therefore participants as well as the trial clinicians were not blinded for the treatment, the outcome assessors (histology and PCR) were blinded for the treatment allocation.

Primary outcome of this trial was the absence of *Wolbachia* endobacteria in adult living female worms assessed by immunohistology 6 months after treatment. Secondary outcomes were the quantitative evaluation of *Wolbachia* endobacteria reduction after 6 months by immunohistology as well as by PCR. Furthermore reduction and presence or absence of Mf in the skin and embryogenesis within the worms as well as the number of live or dead worms was evaluated.

### Parasitological assessment

All infected patients presented at least one palpable nodule [32]. For Mf analysis, two skin biopsies of 1–3 mg were taken from the buttocks using a corneoscleral (Holth) punch. Each biopsy was immersed in 100 ml of 0.9% NaCl solution in a well of a microtiter plate. The skin biopsies were incubated overnight at room temperature to allow Mf to emerge. The solution was then transferred onto a slide for microscopic examination [15, 33]. The biopsies were weighed using an electronical balance and Mf load was calculated per mg skin. The presence of further parasitic infections were assessed using standard diagnostic tests on stool and urine samples [34]. Five participants were positive for hookworm, one for *Giardia lamblia* and one for *Strongyloides*.

### Histological assessment

Nodules were fixed in 80% ethanol or 4% phosphate buffered formaldehyde solution. Samples were embedded in paraffin and several sections were stained with hematoxylin and eosin, Gomori’s method for iron, or immunostained using antibodies against *Dirofilaria immitis Wolbachia* surface protein (Diwsp) or *Wolbachia* PAL-lipoprotein (*wBm*PAL) for presence of *Wolbachia* and cathepsin D-like lysosomal aspartic protease of *O*. *volvulus* (APR) for worm vitality [35]. Thereby at least 8 sections across the nodules were histologically assessed as described previously [36, 37]. In brief, characteristics for death of a worm included loss of body wall integrity, loss of nuclei of all organs and absence of APR-staining. Very degenerated worms, still APR-positive, were classified as moribund and grouped in the category “dead”. The designation “living” refers to worms judged as being alive at the time of nodulectomy. Characteristics for differentiation include the size, general organ or tissue structure as well as iron deposition in the intestine of the worm. The age of the living worms was estimated as "newly acquired", “young”, “middle”, “old” (see Fig 2) [37]. Thirteen male worms were defined as newly acquired. They were not subtracted from this analysis because these worms could have been acquired already shortly before treatment onset. The classification of *Wolbachia* content was: no *Wolbachia* (negative for Diwsp or *wBm*PAL staining), few or many. Since Wolbachia are densely packed within the hypodermis, the female worms containing such conglomerates were assigned as many, whereas those female worms containing visible *Wolbachia* (<50) but not in dense packages were assigned as few (see Fig 2). Developmental stages from the two-cell stage through to stretched Mf in the uterus were classified as “embryos”. When only the stretched Mf were degenerated and the other embryo stages were not, embryogenesis was recorded as “normal” for the purpose of this tetracycline-oriented study, since the previous IVM treatment would have conferred precisely these effects, i.e. degeneration of intra-uterine Mf (in addition to killing of skin Mf). The sections were assessed by two experienced parasitologists (SS, BD).

### PCR

For DNA extraction 8 nodule paraffin sections of 4 μm were placed in microcentrifuge tubes and DNA was extracted according to the manufacturer’s instructions (Quiamp DNA Mini Kit). The *Wolbachia ftsZ* and *O*. *volvulus actin* gene were quantified from the purified DNA by real-time duplex PCR (qPCR) using the following conditions: 10x HotStar Taq Polymerase buffer (Qiagen), 200 μM dNTP, Primers: OvFtsZ forward primer (aggaatgggtggtggtactg), OvFtsZ reverse primer (ctttaaccgcagctcttgct), OvActin forward primer (gtgctacgttgctttggact), OvActin reverse primer (gtaatcacttggccatcagg), OvFtsZ taqman probe (5’6-FAM ccttgccgctttcgcaatcac 3’DDQ-1), OvActin taqman probe(5’HEX aacaggaaatggcaactgctgc 3’BHQ-1), 2.5 units HotStar Taq, and 2 μl DNA in a 20 μl reaction. Final primer concentrations were 400 nM for OvwFtsZ and OvActin, final taqman probe concentrations were 25 nM for OvwFtsZ and 50 nM for OvActin and 6 mM for the MgCl_2_ concentration. Genes were amplified in a Rotorgene 3000 (Quiagen, Hilden, Germany) using the following conditions: 1X 15 min at 95°C, 45 cycles of 95°C for 15 sec, 58°C for 30 sec. Fluorescence was acquired on the FAM and JOE channel.

### Statistics

Analyses were done using SPSS (IBM SPSS Statistics 22; Armonk, NY) and SAS version 9.2 (SAS Institute Inc. Cary, NC, USA).

**Fig 1:**
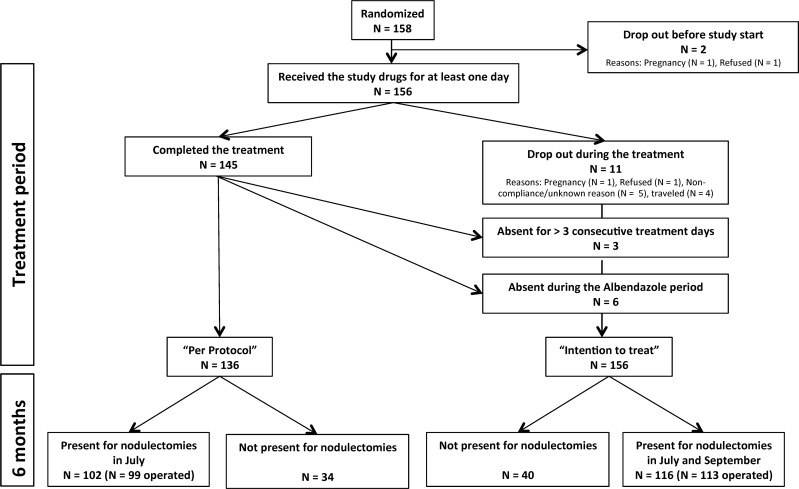
Flow chart of volunteers who took part in the study.

Three data sets (per protocol, PP; intention to treat, ITT; Mf-PP) were used to analyze the data (Fig 1). The intention to treat set (ITT) includes all patients who were randomized to one of the 5 treatment regimens and who took the drugs at least once. The PP set is a subset of the ITT set and includes all patients who completed the treatment without any violations of the protocol and were present for nodulectomies 6 months after treatment onset. Since this is a non-confirmatory pilot-trial all endpoint analyses were primarily carried out using the PP set. The ITT set was used to describe the baseline data, the adverse events and for confirmation of the PP analyses. For the ITT outcome analyses of the histological and PCR results we included all operated individuals, missing values were not replaced. Additionally, we set up a data set, including all patients present for skin-snipping before treatment onset as well as at the 6 months follow-up (Mf-PP).

**Fig 2:**
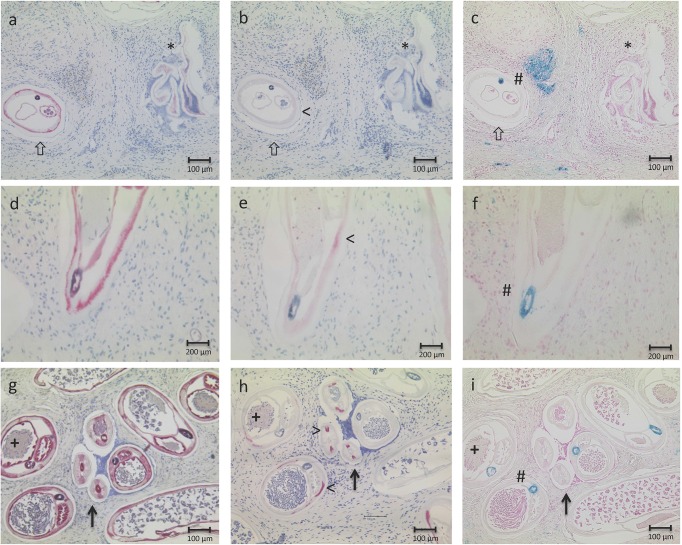
Histological analysis of endobacteria content and worm age. Worms were categorized using three different staining methods, i.e. APR (a, d, g), anti-Diwsp or *wBm*PAL (b, e, h) and iron staining (c, f, i). Worms were alive at the time of nodulectomy (a, d, g), when they stained positive for APR in the hypodermis, embryos, gut and uteri, whereas dead worms were APR-negative (a, *). *Wolbachia* levels were categorized as negative (b, <), few (e, <) or many (h, <). In addition, age of the worms was estimated as described previously and included a combination of size, general organ and tissue structure (a, b, d, e, g, h) as well as iron deposition in the gut (#, c, f, i). Worms shown here were classified as old (> a-c), middle aged (d, e, f), young (+, >, g, h, i).

Alternating regression, as implemented in the SAS-Procedure Genmod, was used to analyze the histological and PCR data. This procedure allows accounting for a potential dependency between the worms in one patient. Odds ratios (with 95% confidence intervals (CIs)) were derived from the regression models to depict the difference between treatment groups with respect to the outcome measurements (*Wolbachia* depletion, inhibition of embryogenesis, occurrence of dead worms, FtsZ, actin, FTsZ/actin). Except for the variable "treatment groups", no covariates were included as effects in the primary analyses. Quantitative PCR data were log_10_-transformed before testing. Comparisons between histological outcome and PCR were done using the unpaired t-test for dead vs. live and ANOVA followed by unpaired t-test for no, many, few *Wolbachia* as well as ANOVA followed by Tukey’s method as post-hoc test for comparison of embryogenesis.

For the analysis of Mf, the Wilcoxon-signed-rank-test was used for comparisons within the patients before and after therapy and the Kruskal-Wallis-test for comparisons between all groups. Baseline data were analyzed using ANOVA for patient age and weight, Fisher’s exact test for gender and the Kruskal-Wallis test for number of nodules and sites.

## Results

### Participant flow and recruitment

The trial profile of the study is illustrated in Fig 1. Altogether, 158 volunteers from 14 villages affected by onchocerciasis that met the inclusion criteria were enrolled into the study and subsequently randomized into the following treatment arms: doxycycline 4 weeks (DOX 4w), doxycycline 3 weeks (DOX 3w), doxycycline 3 weeks plus albendazole 3 days (DOX 3w + ALB 3d), minocycline 3 weeks (MIN 3w) or albendazole 3 days (ALB 3d) as equivalent to a negative control (see Materials and Methods). Baseline data for the participants who took the drugs for at least one day (ITT) with regard to gender, age, weight, and rounds of IVM of the volunteers in each treatment group are given in Table 1. On average, participants had two palpable nodules and one round of IVM prior to treatment begin. 39% of the study participants had never taken IVM. Mf positivity and the intensity of microfilaridermia (Mf/mg skin) did not differ between the groups at study onset with 63.6% Mf-positive individuals.

**Table 1:** Patient baseline data

				Treatment				
			Total	DOX 4w	DOX 3w+ ALB 3d	MIN 3w	DOX 3w	ALB 3d
Age (years)	N		156	33	32	30	31	30
	Mean ± SD		40.1 ± 10.0	40.6 ± 9.9	40.4 ± 10.3	41.0 ± 10.2	37.2 ± 9.4	41.6 ± 10.4
				*p* = 0.4521^a^				
Weight (kg)	N		156	33	32	30	31	30
	Mean ± SD		58.3 ± 7.7	59.0 ± 7.1	57.7 ± 8.5	58.7 ± 8.7	57.4 ± 6.6	58.9 ±7.5
				*p* = 0.8812^a^				
Sex	female		63 (40.4%)	9 (27.3%)	14 (43.8%)	11 (36.7%)	14 (45.2%)	15 (50.0%)
	male		93 (59.6%)	24 (72.7%)	18 (56.3%)	19 (63.3%)	17 (54.8%)	15 (50.0%)
				*p* = 0.3859^b^				
IVM Rounds	N		156	33	32	30	31	30
	Mean ± SD		1.0 ± 1.0	0.8 ± 0.9	1.0 ± 1.1	1.1 ± 1.0	1.2 ± 1.1	0.9 ±1.0
	Min—Max		0–5.0	0–3.0	0–5.0	0–3.0	0–3.0	0–3.0
		Median	1.0	1.0	1.0	1.0	1.0	1.0
		Percentiles 25^th^;75^th^	1.0–2.0	0–1.0	0–2.0	0–2.0	0–2.0	0–1.0
				*p* = 0.4222^c^				
	0		60 (38.5%)	15 (45.5%)	13 (40.6%)	10 (33.3%)	9 (29.0%)	13 (43.3%)
	1		52 (33.3%)	11 (33.3%)	10 (31.3%)	9 (30.0%)	11 (35.5%)	11 (36.7%)
	2		29 (18.6%)	5 (15.2%)	7 (21.9%)	8 (26.7%)	6 (19.4%)	3 (10.0%)
	3		14 (9.0%)	2 (6.1%)	1 (3.1%)	3 (10.0%)	5 (16.1%)	3 (10.0%)
	5		1 (0.6%)	0	1 (3.1%)	0	0	0
No. of Nodules	N		156	33	32	30	31	30
	Mean ± SD		2.6 ± 1.7	2.6 ± 1.9	2.7 ± 2.0	2.6 ± 1.7	2.8 ± 1.5	2.4 ± 1.3
	Median		2.0	2.0	2.0	2.0	3.0	2.0
				*p* = 0.7858^c^				
No. of Sites	N		156	33	32	30	31	30
	Mean ± SD		1.8 ± 0.9	1.7 ± 1.1	1.9 ± 1.2	1.6 ± 0.8	1.8 ± 0.8	1.7 ± 0.7
	Median		2.0	1.0	2.0	1.0	2.0	2.0
				*p* = 0.5999^c^				

^a^ ANOVA

^b^ Fisher's Exact Test

^c^ Kruskal-Wallis Test

Since already 8 participants dropped out before (N = 2) or during the first 2 treatment days (N = 6), 8 additional participants were randomized to avoid a higher drop-out rate than primarily calculated. From 156 participants who had started treatment, 116 (73.4%) presented for nodulectomy (109 at the 6 months follow-up and 7 two months later); of these, 99 participants could be included in the PP set (Fig 1), as they had completed treatment according to protocol, i.e. had not been absent for more than 3 consecutive days or during the ALB period (treatment days 9–11).

### Development of adverse reactions

During treatment, adverse reactions (AR) to the study drugs were monitored. The analyses showed that although significant differences occurred between the study groups (S1 and S2 Tables), the adverse reactions reported were minor (Grade 1 or Grade 2, only once Grade 3) and included stomach pain, dizziness, vomiting and nausea. Most of the reported AR occurred in the combination group DOX 3w + ALB 3d, however AR were not associated with the time point of ALB intake. The number and type of AR were similar in the DOX 4w and DOX 3w group, therefore a longer treatment time does not alter AR. It is important to note that the range of AR after DOX is similar to that seen in a previous study [10], in which DOX was used in lower doses and was performed under blinded conditions. MIN associated AR were mainly dizziness, a common side effect of this drug.

Taken together, the study drugs were well tolerated and no severe or unexpected events occurred due to intake of the medications.

### Immunohistology

#### Depletion of *Wolbachia*

As primary outcome, the presence of *Wolbachia* endobacteria in worm tissues was examined by immunohistology. All histological analyses were performed PP as well as ITT (supplementary data).

In total, 272 nodules from 98 individuals (PP, Tables 2–4) and 307 nodules from 110 study participants (ITT, S3–S5 Tables) were examined. 8.7% of all nodules (PP; ITT: 10.8%) could not be analysed for viability status; the reasons for this included being of non-onchocercal origin (e.g. foreign body granulomas, lipomas or lymph nodes).

**Table 2:** Effect of the study drugs on presence of *Wolbachia* in female worms: histology

Treatment Group	No. of Patients/ Nod ^a^	No. of living female worms			
		All	*Wolbachia* levels		
	98 / 272	334	many	few	none
DOX 4w (Standard)	24 / 62	80	0	1 (1.3%)	79 (98.8%)
DOX 3w + ALB 3d	18 / 54	70	0	13 (18.6%)	57 (81.4%)
MIN 3w	19 / 46	66	0	18 (27.3%)	48 (72.7%)
DOX 3w	18 / 46	64	1 (1.6%)	22 (34.4%)	41 (64.1%)
ALB 3d	19 / 64	54	5 (9.3%)	30 (55.6%)	19 (35.2%)

^a^ Only evaluable patients/nodules are included.

**Table 3:** Effect of the study drugs on presence of *Wolbachia* in male worms: histology

Treatment Group	No. of Patients/ Nod ^a^	No. of living male worms			
		All^b^	*Wolbachia* levels		
	98 / 272	119	many	few	none
DOX 4w (Standard)	24 / 62	25	0	3 (12.0%)	22 (88.0%)
DOX 3w + ALB 3d	18 / 54	23	3 (13.0%)	4 (17.4%)	16 (69.6%)
MIN 3w	19 / 46	23	4 (17.4%)	4 (17.4%)	15 (65.2%)
DOX 3w	18 / 46	25	3 (12.0%)	12 (48.0%)	10 (40.0%)
ALB 3d	19 / 64	23	2 (8.7%)	11 (47.8%)	10 (43.5%)

^a^ Only evaluable patients/nodules are included.

**Table 4:** Effect of the study drugs on presence of *Wolbachia* in female worms: statistics^a^^,^ ^b^

PP, female	DOX 3w + ALB 3d	MIN 3w	DOX 3w	ALB 3d
DOX 4w	***p* = 0.0052OR 17.98 [2.37; 136.1]**	***p* = 0.0022OR 25.97 [3.24; 208.33]**	***p* = 0.0007OR 36.24 [4.55; 288.84]**	***p*<0.0001OR 145.0 [18.8; 118.08**
DOX 3w + ALB 3d		*p* = 0.5538OR 1.36 [0.47; 4.08]	*p* = 0.2607OR 1.85 [0.63; 5.44]	***p*<0.0001OR 8.16 [3.08; 21.57]**
MIN 3w			*p* = 0.5981OR 1.36 [0.43; 4.28]	***p* = 0.0016OR 5.76 [1.95; 17.03]**
DOX 3w				***p* = 0.0084OR 4.2 [1.44; 12.2]**

^a^ Alternating logistic regression (comparison of presence (many and few) vs. absence of *Wolbachia*)

^b^ Table shows the odds ratios (OR) for presence of *Wolbachia* comparing the treatment groups in the headline to the treatment groups in the left column.

65% of the female worms in the control group (ALB 3d) were *Wolbachia*-positive (Table 2), a level that is consistent with other endemic areas with lowered transmission and a higher proportion of older worms [10], possibly induced by MDA or as a result of intensified gold digging and thus pollution of the water breeding habitat of the vector [38]. In contrast, only 1.3% contained intact *Wolbachia* in the standard treatment group (DOX 4w). A similar difference could be observed for male worms, with 56.5% *Wolbachia* positive worms in the ALB 3d group and 12% in the standard DOX 4w group, respectively (Table 3).

All experimental treatment groups (DOX 3w+ALB 3d (OR 8.16 [3.1; 21.6], *p*< 0.0001), MIN 3w (OR 5.8 [2.0; 17.0], *p* = 0.0016) and DOX 3w (OR 4.2 [1.4; 12.2], *p* = 0.0084) showed superiority to the ALB 3d group when using alternating logistic regression. However, the extent of *Wolbachia* depletion was superior in the DOX 4w group (OR 145 [18.8; 118.1], *p*< 0.0001), compared to all experimental treatment groups as well as to ALB 3d (OR 145 [18.8; 118.1], *p*< 0.0001; Table 4). Despite this finding, the DOX 3w+ALB 3d (81.4%) and the MIN 3w (72.7%) arms showed higher *Wolbachia* depletion compared to DOX 3w (64.1%) indicating a stronger efficacy. Furthermore, the ORs presented in Table 4 suggest that there is a possible synergistic effect of DOX 3w+ALB 3d compared to DOX 3w alone (OR 1,85 [0.63; 5.44]), albeit not to a statistically significant degree (*p* = 0.2607). Under ITT conditions both groups (DOX 3w+ALB 3d and MIN 3w) were not statistically different to DOX 4w (S3–S5 Tables). This however does not imply their equivalence to the DOX 4w group, as the ORs 3.7 [0.6; 22] and 5.1 [0.8; 32] still indicate a higher risk for presence of *Wolbachia* in both groups. In male worms, *Wolbachia* were reduced significantly in the DOX 4w group compared to the ALB 3d control group (OR 7.9 [1.7; 37.3], p = 0.0093) and to DOX 3w (OR 10.1 [2.2; 46.3], p = 0.0031).

To exclude the possibility that prior MDA participation may have influenced *Wolbachia* depletion, the alternating logistic regression was additionally performed with prior IVM-rounds as single effect variable showing no effect on the outcome (*p* = 0.7932). Including prior IVM rounds as covariate in the treatment model revealed no relevant influence on the treatment effects described above.

All these findings indicate a trend towards a better efficacy of DOX 3w + ALB 3d or MIN 3w compared to DOX 3w but designed as a non-confirmatory pilot trial the study was not planned to detect such subtle differences. A larger confirmatory trial setting would be needed to identify whether DOX 3w + ALB 3d or MIN 3w are better than DOX 3w.

In summary, the data shown here provide evidence that all treatments are superior to ALB in their ability to deplete *Wolbachia* with DOX 4w showing the strongest efficacy.

#### Inhibition of embryogenesis

As a secondary endpoint of the study, we investigated the drug-induced blockade of worm embryogenesis, being defined as the proportions of worms with normal or degenerated embryogenesis. For this analysis, we considered the different activities of DOX, ALB and IVM on embryogenesis. IVM has been demonstrated to lead to degeneration of stretched Mf in the uterus whilst there is limited to no effect on the morulae and coiled Mf stages [14]. In contrast, anti-wolbachial therapy inhibits the early stages of embryogenesis. Although DOX does not affect fully developed Mf, neither in utero nor after release when free in the host’s tissues, there is evidence that they may be inhibited from developing within the vector [39]. ALB has only minor effects on embryonic stages of *O*. *volvulus* [31, 40, 41]. We found that in the ALB 3d group 17.7% of all 54 female worms analyzed had normally developed embryos (Table 5; S6 and S7 Tables) and 12.8% of the nodules were Mf-positive (Table 7, S8 Table). In contrast, in the DOX 4w group only 8% of all 75 female worms contained normal embryos and all nodules were Mf-negative. Similarly, only 14.7% of 68 female worms analyzed in the DOX 3w+ALB 3d group showed normal embryogenesis with 8.7% Mf-positive nodules. Comparison of DOX 4w and DOX 3w plus Alb 3d to Alb 3d alone using alternating logistic regression showed a higher risk for the Alb 3d group to maintain living female worms with normal embryogenesis compared to worms with degenerated embryogenesis (OR 4 [1.2; 13.8], *p* = 0.0283; OR 4.8 [1.1; 21.5], *p* = 0.0423, respectively, Table 6). A trend for reduction of normal embryogenesis within female worms was also seen in the MIN 3w group (11.7%) and, to a lesser extent, in the DOX 3w group with 17.7% females showing normal embryogenesis in 62 female worms analyzed.

**Table 5:** Effect of the study drugs on embryogenesis: histology

Treatment Group	No. of Patients/Nod ^a^	No.of living female worms					
		All	Embryos				Sperms in Uterus
	98 / 272	334	not judgeable	oocytes only / uterus empty	normal	degenerated	
DOX 4w (Standard)	24 / 62	80	5	57 (76.0%)	6 (8.0%)	12 (16.0%)	10 (12.5%)
					(33.3%)^b^	(66.7%)^b^	
DOX 3w + ALB 3d	18 / 54	70	2	42 (61.8%)	10 (14.7%)	16 (23.5%)	14 (20.0%)
					(38.5%)^b^	(61.5%)^b^	
MIN 3w	19 / 46	66	6	41 (68.3%)	7 (11.7%)	12 (20.0%)	7 (10.6%)
					(36.8%)^b^	(63.2%)^b^	
DOX 3w	18 / 46	64	2	42 (67.7%)	11 (17.7%)	9 (14.5%)	10 (15.6%)
					(55.0%)^b^	(45.0%)^b^	
ALB 3d	19 / 64	54	3	39 (76.7%)	9 (17.7%)	3 (5.9%)	9 (16.7%)
					(75.0%)^b^	(25.0%)^b^	

^a^ Only evaluable patients/nodules are included.

^b^ % of all female worms with embryogenesis within the respective group

**Table 6:** Effect of the study drugs on embryogenesis: statistics^a^

PP	DOX 3w + ALB 3d	MIN 3w	DOX 3w	ALB 3d
DOX 4w	*p* = 0.7862OR 1.16 [0.41;3.3]	*p* = 0.7245OR 1.28 [0.32;5.13]	*p* = 0.0571OR 1.98 [0.98;4.0]	***p* = 0.0283OR 4.0 [1.16;13.83]**
DOX 3w + ALB 3d		*p* = 0.937 OR 0.94 [0.23;3.87]	*p* = 0.2417OR 1.96 [0.64;6.06]	***p* = 0.0423OR 4.77 [1.06;21.54]**
MIN 3w			*p* = 0.3613OR 2.0 [0.45;8.89]	*p* = 0.1182 OR 4.07 [0.7;23.7]
DOX 3w				*p* = 0.2149 OR 2.51 [0.59;10.77]

^a^ alternating logistic regression, comparison of normal vs. degenerated embryogenesis

^b^ Table shows the odds ratios (OR) for embryogenesis comparing the treatment groups in the headline to the treatment groups in the left column.

**Table 7:** Effect of the study drugs on Mf within the nodule: histology

Treatment Group	No. of Patients/Nod^a^	No. of Nodules^b^	
		All	with intact mf
	98 / 272	208	
DOX 4w	24 / 62	53	0
DOX 3w + ALB 3d	18 / 54	46	4 (8.7%)
MIN 3w	19 / 46	38	2 (5.3%)
DOX 3w	18 / 46	32	2 (6.3%)
ALB 3d	19 / 64	39	5 (12.8%)

^a^ Only evaluable patients/nods are included

^b^ Only nodules with living female worms are included

To examine a possible influence of the prior IVM round on the embryogenesis, the alternating logistic regression was additionally performed with prior IVM-rounds as single effect variable showing a tendency towards normal embryogenesis versus degenerated embryogenesis in patients with fewer IVM-rounds (OR 1.5 [0.96; 2.24], *p* = 0.0745). But, including prior IVM-rounds as covariate in the treatment model revealed again no relevant influence on the treatment effects described above.

In summary, the data show that a combination of DOX 3w with ALB 3d leads to small but detectable additive effects in depleting *Wolbachia* and thereby probably increased inhibition of embryogenesis in both data sets (Tables 5–7 and S6–S8 Tables). Furthermore, the partial effect of MIN 3w compared to DOX 3w suggests that when used for longer treatment times, minocycline is more efficacious than doxycycline.

#### Effect on adult worms

The aim of this study was the investigation of *Wolbachia* absence at 6 months post treatment to identify drug regimens with faster–acting antibiotic activity. *Wolbachia* reduction has been shown to precede macrofilaricidal activity and earlier studies by our group have shown that an observation period of 20 and 27 months is needed in order to observe macrofilaricidal effects [15]. Still, we analyzed the number of dead worms in the different treatment groups. 41.8% dead females and 4.0% dead males were found at 6 months post treatment. There were no significant differences between the study groups. On average, each nodule contained a total of 2.11 females (1.23 living females, 0.88 dead females) and 0.46 males (0.44 living males, 0.02 dead males).

### Real-time PCR

#### Depletion of *Wolbachia*

Real-time polymerase chain reaction is a very sensitive technique that allows the quantification of a given DNA segment. We wanted to investigate whether PCR detection of *Wolbachia* DNA confirms the results as read by immunohistology and thereby offers an option for quantification of *Wolbachia* levels directly from histological sections, leaving the nodule otherwise intact for further histological investigations. Therefore, DNA from 8 sections adjacent to the ones analyzed with histology were chosen and analyzed for *Wolbachia* FtsZ and nematode actin. We were able to isolate DNA from all but 5 nodules that were analyzed with immunohistology (see above).

Importantly, the FtsZ as well as the FtsZ/actin ratio (Table 8) confirm the immunohistology results and similar gradations occurred between the groups and the control group, with DOX 4w weeks showing the strongest reduction, followed by DOX 3w + ALB 3d, MIN 3w and DOX 3w in the PP data set (Tables 9–11) as well as the ITT dataset (S9–S12 Tables). Actin levels did not differ between the groups, since nodules with dead or resorbed worms only were subtracted prior to this statistical analysis.

**Table 8:** Effect of the study drugs on presence of *Wolbachia* in nodule sections: PCR^a^^,^^b^

		DOX 4w	DOX 3w + ALB 3d	MIN 3w	DOX 3w	ALB 3d
FtsZ	N	53	47	39	36	41
	Median	410	960	3870	2990	8460
	95% CI (Median)	128;933	485;3260	580;5790	1000;6425	5210;13500
	Percentiles 25^th^;75^th^	75;2310	163;6030	278;8430	369;13550	2840;28100
	Min—Max	0.06–25600	0.21–195000	0.03–99000	0.00–131000	0.27–130000
Actin	N	53	47	39	36	41
	Median	1170	2020	981	1365	1230
	95% CI (Median)	933;2239	901;3810	448;2430	413;3030	650;1875
	Percentiles 25^th^;75^th^	470;3190	638;6340	354;5480	204;5743	262;3190
	Min—Max	4.30–27900	11.70–48600	9.18–405000	1.94–114000	37.30–45300
FtsZ/Actin	N	53	47	39	36	41
	Median	0.25	0.51	1.23	1.36	11.14
	95% CI (Median)	0.09;0.79	0.42;0.82	0.4;2.72	1.06;4.03	2.09;23.13
	Percentiles 25th;75th	0.03;1.60	0.22;2.63	0.20;6.24	0.66;5.35	1.40;36.75
	Min—Max	0–27.44	0–45.03	0–250.54	0–79.03	0–2832.24

^a^ Normally one PCR per nodule (from 3 nodules 2 PCRs were done and the mean was taken for further analyses (DOX 4w N = 2, DOX 3w N = 1), in 1 nodule PCR could not be performed (DOX 4w))

^b^ For the analyses, PCRs from all nodule with dead worms only were excluded from this table and from all following analyses

**Table 9:** Effect of the study drugs on presence of *Wolbachia* in nodule sections: statistics for FtsZ^a^^,^^b^

PP	DOX 3w + ALB 3d	MIN 3w	DOX 3w	ALB 3d
DOX 4w	*p* = 0.0954	***p* = 0.0403**	*p* = 0.061	***p*<0.0001**
	OR 3.05 [0.82;11.35]	**OR 4.51 [1.07;19.05]**	OR 4.3 [0.94;19.73]	**OR 22.46 [6.99;72.15]**
DOX 3w + ALB 3d		*p* = 0.5455	*p* = 0.6702	***p* = 0.0006**
		OR 1.54 [0.38;6.21]	OR 1.38 [0.32;6.02]	**OR 6.88 [2.3;20.59]**
MIN 3w			*p* = 0.9193	***p* = 0.0168**
			OR 0.92 [0.19;4.55]	**OR 4.68 [1.32;16.59]**
DOX 3w				***p* = 0.0171**
				**OR 5.11 [1.34;19.52]**

^a^ Alternating linear regression (after log_10_-transformation (all values +0.1 to circumvent zero values))

^b^ Table shows the odds ratios (OR) for presence of *Wolbachia* comparing the treatment groups in the headline to the treatment groups in the left column.

**Table 10:** Effect of the study drugs on presence of *Wolbachia* in nodule sections: statistics for Actin^a^

PP	DOX 3w + ALB 3d	MIN 3w	DOX 3w	ALB 3d
DOX 4w	*p* = 0.3888	*p* = 0.6398	*p* = 0.6897	*p* = 0.8569
	OR 1.43 [0.64;3.2]	OR 1.25 [0.49;3.2]	OR 0.79 [0.25;2.53]	OR 0.93 [0.4;2.15]
DOX 3w + ALB 3d		*p* = 0.8214	*p* = 0.3758	*p* = 0.3281
		OR 0.89 [0.34;2.35]	OR 0.58 [0.18;1.92]	OR 0.65 [0.27;1.55]
MIN 3w			*p* = 0.5971	*p* = 0.6193
			OR 0.71 [0.2;2.5]	OR 0.78 [0.29;2.1]
DOX 3w				*p* = 0.734
				OR 1.23 [0.37;4.12]

^a^ Alternating linear regression (after log_10_-transformation (all values +0.1 to circumvent zero values)) ^b^ Table shows the odds ratios (OR) for presence of *Wolbachia* comparing the treatment groups in the headline to the treatment groups in the left column.

**Table 11:** Effect of the study drugs on presence of *Wolbachia* in nodule sections: statistics for FtsZ/Actin^a^^,^^b^

PP	DOX 3w + ALB 3d	MIN 3w	DOX 3w	ALB 3d
DOX 4w	*p* = 0.3403	***p* = 0.0168**	***p* = 0.0036**	***p*<0.0001**
	OR 1.43 [0.69;2.97]	**OR 2.63 [1.19;5.81]**	**OR 3.07 [1.44;6.54]**	**OR 11.69 [4.88;28.02]**
DOX 3w + ALB 3d		*p* = 0.2097	*p* = 0.0634	***p*<0.0001**
		OR 1.68 [0.75;3.78]	OR 2.05 [0.96;4.39]	**OR 7.66 [3.18;18.47]**
MIN 3w			*p* = 0.6526	***p* = 0.0004**
			OR 1.19 [0.55;2.57]	**OR 4.88 [2.02;11.81]**
DOX 3w				***p* = 0.0017**
				**OR 4.04 [1.69;9.69]**

^a^ Alternating linear regression (after log_10_-transformation (all values +0.1 to circumvent zero values))

^b^ Table shows the odds ratios (OR) for presence of *Wolbachia* comparing the treatment groups in the headline to the treatment groups in the left column.

The lack of complete absence of *Wolbachia* for example in the DOX 4w group, may result from an inability of the worm to degrade the *Wolbachia* DNA within 6 months, whereas their bacterial function and histological visibility is already lost.

#### Comparison of immunohistology and real-time PCR

**Fig 3:**
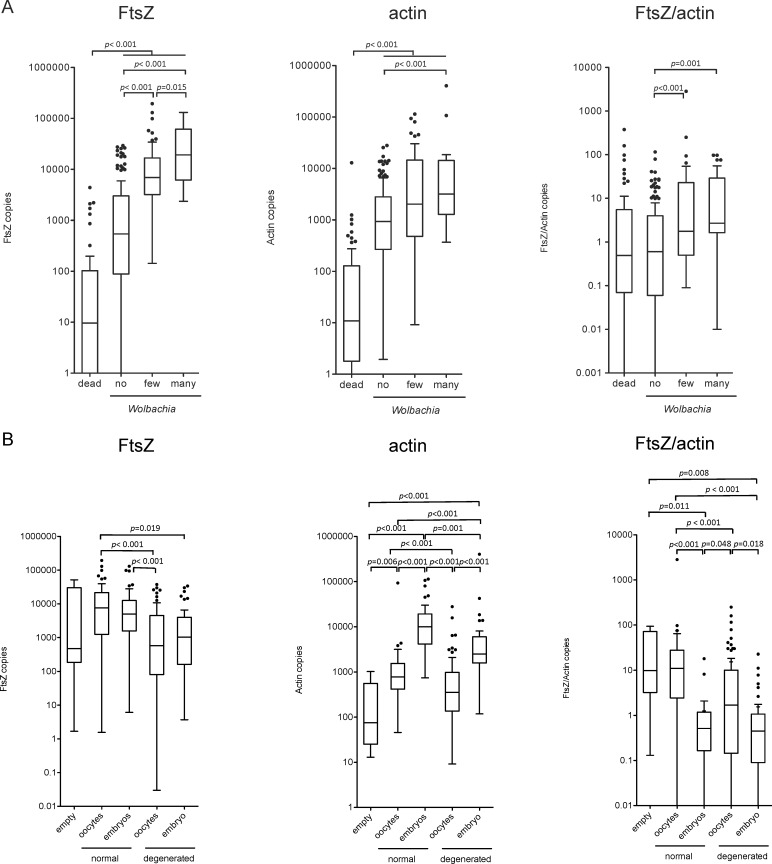
Comparison of immunohistology and real-time PCR (*Wolbachia* depletion and embryogenesis). FtsZ, actin copy numbers and the FtsZ/actin ratio are shown. (A) Data were split by their immunohistological outcome (*Wolbachia* content, live vs. dead worms) and significances were calculated for 1) nodules containing dead worms only vs. all nodules containing live worms and 2) nodules containing live worms separated into no, few or many *Wolbachia*. (B) Data were split by their immunohistological outcome (embryogenesis). The overall comparisons regarding embryogenesis showed a significant correlation for FtsZ, actin and FtsZ/actin copies (*p*< 0.001, respectively; ANOVA). Tukey’s method was used for post-hoc comparisons.

Both, FtsZ and actin may be strongly influenced by the numbers of worm sections within a sample, the presence of oocytes or further developed stages within the female worms or whether the worms found in the nodule sections are alive or dead. Therefore we subdivided the *Wolbachia* depletion data according to the histological differentiation of i) dead worms vs. live worms and to ii) live worms with no, few or many *Wolbachia* (Fig 3A). Accordingly, the *Wolbachia* signal FtsZ and the filarial actin were low in nodules containing dead worms only compared to nodules containing also live worms (p < 0.001 ANOVA). FtsZ, actin as well as the FtsZ/actin ratio reflected the increase of *Wolbachia* showing significant differences between no and few or no and many *Wolbachia*. Furthermore, a difference between few and many *Wolbachia* could be detected by the *Wolbachia* signal FtsZ (*p* = 0.015).

In addition, grouping by embryogenesis showed a relation of the FtsZ to the normal development of oocytes and embryos in the female worm. The actin level revealed a strong dependency from the presence of oocytes or embryos within the uterus of the females, reflecting the increased amount of nuclei with offspring production (Fig 3B).

Thus, the PCR performed on nodule tissue section reflected the histological evaluation.

#### Effect on microfilaridermia

Of the 102 study participants who completed the treatment per protocol and were present for skin snipping after 6 months, 65 (63.7%) were microfilaridermic (Mf-positive) at entry to the study, with the remaining 37 (36.3%) having no detectable skin microfilariae but only palpable nodules before treatment. In accordance to our earlier study [35], at the 6-month follow-up time point, no significant differences in the number of Mf-carriers were observed between the groups themselves and compared to pretreatment, indicating that this time point is too early to observe study treatment-induced reduction of peripheral Mf (Table 12; S13 Table). Similarly, there was no significant difference in the Mf load (median Mf/mg skin) between all treatment groups prior to treatment (Table 13; S14 Table). And, also after 6 months, no differences between the groups occurred. However, a statistically significant decrease in the ALB 3d group (*p* = 0.047, median pre-treatment 0.4, median 6 months 0.2) and a trend in the DOX 4w group (*p* = 0.091, median pre-treatment 2.3, median 6 months 0.7) was observed. Both results could be confirmed by the ITT analysis (ALB 3d: *p* = 0.025, DOX 4w: *p* = 0.028).

**Table 12:** Microfilaridermia

	Pre-treatment		6 months	
	Mf-positive	Mf-negative	Mf-positive	Mf-negative
DOX 4w	16 (66.7%)	8 (33.3%)	16 (66.7%)	8 (33.3%)
DOX 3w + ALB 3d	11 (61.1%)	7 (38.9%)	13 (72.2%)	5 (27.8%)
MIN 3w	13 (61.9%)	8 (38.1%)	12 (57.1%)	9 (42.9%)
DOX 3w	12 (63.2%)	7 (36.8%)	11 (57.9%)	8 (42.1%)
ALB 3d	13 (65.0%)	7 (35.0%)	12 (60.0%)	8 (40.0%)

**Table 13:** Levels of Mf

		Treatment				
		DOX 4w	DOX 3w	DOX 3w + ALB 3d	MIN 3w	ALB 3d
Pre-treatment^c^	N	24	19	18	21	20
	Mean ± SD	5.3 ± 7.5	6.2 ± 11.2	6.1 ± 10.5	4.7 ± 13.4	4.5 ± 9.7
	GM^a^	2.4	1.8	2.3	1.2	1.4
	Min—Max	0–28.0	0–36.3	0–40.2	0–61.0	0–38.9
	Median	2.3	0.4	2.1	0.3	0.4
	95% CI (median)	0;4.7	0;0.2	0;4.7	0;2.1	0;3.3
	Percentiles 25^th^;75^th^	0; 6.0	0; 7.4	0; 4.8	0; 2.1	0; 4.5
6 months^d^	N	24	19	18	21	20
	Mean ± SD	3.8 ± 7.4	10.1 ± 36.0	4.4 ± 7.8	6.1 ± 14.2	3.0 ± 6.9
	GM^a^	1.5	1.3	1.5	1.4	1.0
	Min—Max	0–33.4	0–157.8	0–21.1	0–48.0	0–29.0
	Median	0.7	0.4	0.7	0.3	0.2
	95% CI (median)	0;2.9	0;1.2	0;1.4	0;3.0	0;1.4
	Percentiles 25^th^;75^th^	0; 4.5	0; 1.3	0; 1.9	0; 4.0	0; 2.2
	*p*-value^b^	*p =* 0.091	*p =* 0.397	*p =* 0.196	*p =* 0.877	***p =* 0.047**

SD = standard deviation, GM = geometric mean

^a^ The geometric mean (GM) was calculated by adding 1 to all values and after the calculation 1 was again subtracted from the result

^b^ Wilcoxon-signed-rank-test

^c^ No difference between all 5 treatment groups pre-treatment (p = 0.799, Kruskal-Wallis-test)

^d^ No difference between all 5 treatment groups at 6 months (p = 0.859, Kruskal-Wallis-test)

## Discussion

Immense efforts have been undertaken in many countries to achieve elimination of onchocerciasis by MDA. However, still 130 million people are at risk of infection [1]. Current approaches to increase the frequency of MDA from annual to biannual treatments are predicted to improve the chances of reaching the 2020/2025 elimination goals at least in some countries. However, its benefits and costs are highly sensitive to systematic noncompliance and it may not always be feasible to implement biannual treatment, particularly in hard-to-reach populations [6, 7, 42]. In addition, given the problem of re-emergence of infections, suboptimal efficacy of IVM [10] and regions that are co-endemic for *Loa loa*, the development of new drugs or drug regimens is urgently needed to achieve the elimination of onchocerciasis. This would increase cost effectiveness by avoiding unnecessary treatments of uninfected individuals within the MDA schemes, especially in “end-game” scenarios or when switching from MDA to “test & treat” strategies.

In this study, we have compared DOX 4w, MIN 3w, DOX 3w plus ALB 3d, DOX 3w and ALB 3d alone in their efficacy against onchocerciasis in a randomized, open-label pilot trial. Since we have shown earlier that *Wolbachia* depletion precedes the inhibition of embryogenesis and adult worm death [14, 15, 43], the follow-up at 6 months post treatment was chosen to investigate the primary outcome, i.e. *Wolbachia* reduction. In the present study, histological analyses of the adult worms within the extirpated nodules 6 months after treatment revealed a *Wolbachia* reduction of 99% in the DOX 4w group showing superiority to all other treatment arms. Accordingly, the extent of *Wolbachia* depletion was highest in the DOX 4w group, followed by DOX 3w + ALB 3d, MIN 3w and then DOX 3w compared to ALB 3d. The *Wolbachia* reduction in the DOX 4w group was comparable to that of a 6-week 200 mg/d regimen with DOX after 6 months [15]. In addition, real-time PCR analysis from DNA isolated from paraffin embedded histological sections revealed that similar gradations occurred between the groups, suggesting the use of real-time PCR as a sensitive method for the quantification of *Wolbachia* loads within the worms after a given treatment. We further validated the FtsZ and actin signal by subdividing the data according to the biological status of the worm (live, dead, *Wolbachia* levels and empty uterus and embryogenesis). FtsZ levels increased according to the histologically defined *Wolbachia* categories, whereas actin levels were elevated, when worms were alive compared to dead worms. In addition, actin levels were associated with increased embryogenesis.

Inhibition of embryogenesis after doxycycline/anti-wolbachial therapy leading to sterile females has been shown to result in the absence of Mf in the skin of infected patients, a requirement for interruption of transmission Therefore, embryogenesis was analyzed as secondary outcome histologically within the nodules as well as by skin MF loads. We found that already after 6 months only 8% (DOX 4w), 14.7% (DOX 3w + ALB 3d), 11.7% (MIN 3w), 17.7% (DOX 3w) of the living female worms with embryogenesis had normal embryonal development compared to 17.7% (ALB 3d). DOX 4w and DOX 3w + ALB 3d showed a higher number of female worms with degenerated embryogenesis compared to ALB 3d and reached statistical significance (OR 4, *p* = 0.0283; OR 4.8, *p* = 0.0423, respectively), whereas MIN 3w and DOX 3w did not. An overall low number of nodules contained MF. However, similar to the results on the absence of *Wolbachia*, the extent of inhibition of normal embryogenesis follows the same order with the highest being in the DOX 4w group, followed by DOX 3w + ALB 3d, MIN 3w and finally DOX 3w. All findings could be confirmed within the ITT analysis.

Other secondary outcomes were microfilaridermia as well as the number of dead worms within the nodules. The 6-months time point is too early for the treatment efficacy to be reflected in the disappearance of peripheral Mf in the skin, since Mf are not killed directly by anti-wolbachial drugs (an advantage e.g. in *Loa* co-endemic areas). In an earlier study, despite the percentage of females with degenerated embryogenesis being increased to 95.5% at 6 months after 6 weeks doxycycline treatment [15], all individuals were still Mf-positive. Similarly, Mf counts were not significantly different between the groups in the present study and 62.7% of the patients were Mf-positive at 6 months after treatment.

Also for the observation of macrofilaricidal efficacy a comparatively long observation period is needed and we have shown that a distinct macrofilaricidal effect can be seen with female worms at 20 and 27 months following a six week treatment course with 200 mg DOX per day [15]. Therefore, similar to the absence of effects on microfilaridermia after 6 months, expectedly no differences could be found in the number of dead worms histologically analyzed within the nodules. Despite this, our data provide clear evidence that the time point of 6 months after treatment can be used for the analysis of *Wolbachia* depletion and in utero inhibition of embryogenesis and is appropriate for identification of anti-wolbachial drugs or drug regimens.

In our study, the 6 months analysis shows that reducing the treatment time with DOX from 4 to 3 weeks is not recommended, as DOX 4w is clearly superior to DOX 3w in *Wolbachia* depletion and inhibition of normal embryogenesis. Therefore a four weeks course of 200 mg DOX should be further recommended to achieve long-term female worm sterility and death [44, 45].

In addition, the combination of 3 weeks of DOX and ALB 3d showed a higher extent of *Wolbachia* depletion compared to 3 weeks of DOX alone, indicating an additive effect when used in combination. Studies to determine whether drug-drug interactions or other mechanisms of action of combinations of anti-*Wolbachia* drugs and anti-filarial drugs lead to enhanced anti-*Wolbachia* and macrofilaricidal effects to further shorten treatment regimens are currently underway as part of the A·WOL programme.

Minocycline, MIN, another broad-spectrum tetracycline antibiotic, was revealed in an *in vitro* assay exposing *O*. *gutturosa* adult males to be more active than DOX [29], as measured by motility of adult worms and viability by reduction of MTT to MTT formazan assay. Since MIN was also among the top hits of the A·WOL screening activities [3], these data were recently confirmed using the *Wolbachia* containing insect cell line C6/36. On top of that, data generated from a rodent infection model using *Litomosoides sigmodontis* confirmed the efficacy *in vivo*, showing a similar superiority of MIN over DOX (Specht et al, in preparation, [30]). Our clinical data reported here support that MIN may actually be more efficacious than DOX, since MIN showed a trend for being better than DOX 3w in the relevant comparisons, i.e. absence of *Wolbachia*, inhibition of normal embryogenesis. The differences did however not reach statistical significance, as the pilot trial design was not sufficiently powered to detect these differences.

In summary, these results provide further evidence and confirm earlier studies [15] that DOX 4w is sufficient for *Wolbachia* depletion and the desired parasitological effects, i.e. depletion of *Wolbachia*, inhibition of embryogenesis, whereas DOX 3w delivers sub-optimal effects. The data presented here further suggest that there is an additive effect of ALB 3d on top of that of DOX 3w alone, since for both outcomes (*Wolbachia* depletion, inhibition of embryogenesis), this group showed the second strongest efficacy after DOX 4w. Furthermore, MIN 3w albeit not significant showed a trend for being more efficacious compared to DOX 3w. This may suggest that when used for longer treatment times, MIN may actually be more efficacious than DOX. These latter two results are preliminary and need confirmation in a full-randomized controlled phase 2 trial.

## Supporting Information

S1 Table Adverse event assessment (DOCX)

S2 Table Adverse event assessment, detailed (DOCX)

S3 Table ITT analysis–Effect of the study drugs on presence of *Wolbachia* in female worms: histology (DOCX)

S4 Table ITT analysis–Effect of the study drugs on presence of *Wolbachia* in male worms: histology (DOCX)

S5 Table ITT analysis–Effect of the study drugs on presence of *Wolbachia* in worms: statistics (c) (DOCX)

S6 Table ITT analysis–Effect of the study drugs on embryogenesis: histology (DOCX)

S7 Table ITT analysis–Effect of the study drugs on embryogenesis: statistics (DOCX)

S8 Table ITT analysis–Effect of the study drugs on Mf within the nodule: histology (DOCX)

S9 Table ITT analysis–Effect of the study drugs on presence of *Wolbachia* in nodule sections: PCR (DOCX)

S10 Table ITT analysis–Effect of the study drugs on presence of *Wolbachia* in nodule sections: statistics FtsZ (DOCX)

S11 Table ITT analysis–Effect of the study drugs on presence of *Wolbachia* in nodule sections: statistics Actin (DOCX)

S12 Table ITT analysis–Effect of the study drugs on presence of *Wolbachia* in nodule sections: statistics FtsZ/Actin (DOCX)

S13 Table ITT analysis–Microfilaridermia (DOCX)

S14 Table ITT analysis–Levels of Mf (DOCX)
