# Supplementary material for: Comparison of Doxycycline, Minocycline, Doxycycline plus Albendazole and Albendazole Alone in Their Efficacy against Onchocerciasis in a Randomized, Open-Label, Pilot Trial
Source: PLoS Negl Trop Dis. 2017 Jan 5;11(1):e0005156. doi: 10.1371/journal.pntd.0005156 (PMC5215804; doi:10.1371/journal.pntd.0005156)
Supplement: S1 Table — (DOCX) [file pntd.0005156.s001.docx]

**S1 table: Adverse event assessment**

|  |  | DOX 4w | DOX 3w + ALB 3d | MIN 3w | DOX 3w | ALB 3d | *p*-value |
| --- | --- | --- | --- | --- | --- | --- | --- |
| No. of patients with AEs |  | 4/33 (12.1%) | 9/32 (28.1%) | 8/30 (26.7%) | 8/31 (25.8%) | 1/30 (3.3%) | **0.048^a^** |
| No. of AEs in total |  | 9 | 16 | 12 | 9 | 1 |  |
| AEs per affected patients | Mean ± SD | 2.25 ± 0.96 | 1.78 ± 0.67 | 1.5 ± 0.76 | 1.13 ± 0.35 | 1 | 0.096^b^ |
|  | Min-Max | 1 - 3 | 1 - 3 | 1 - 3 | 1 - 2 | 1 |  |
|  | Median | 2.5 | 2 | 1 | 1 | 1 |  |
| AEs per total patients | Mean ± SD | 0.27 ± 0.8 | 0.5 ± 0.88 | 0.4 ± 0.77 | 0.29 ± 0.53 | 0.03 ± 0.88 | 0.057^b^ |
|  | Min-Max | 0 – 3 | 0 – 3 | 0 – 3 | 0 – 2 | 0 – 1 |  |
|  | Median | 0 | 0 | 0 | 0 | 0 |  |

^a^ Fisher`s exact test

^b^ Kruskal-Wallis-test
