# Supplementary material for: Comparison of Doxycycline, Minocycline, Doxycycline plus Albendazole and Albendazole Alone in Their Efficacy against Onchocerciasis in a Randomized, Open-Label, Pilot Trial
Source: PLoS Negl Trop Dis. 2017 Jan 5;11(1):e0005156. doi: 10.1371/journal.pntd.0005156 (PMC5215804; doi:10.1371/journal.pntd.0005156)
Supplement: S2 Table — (DOCX) [file pntd.0005156.s002.docx]

**S2 table: Adverse event assessment, detailed**

|  |  |  | DOX 4w | DOX 3w + ALB 3d | MIN 3w | DOX 3w | ALB 3d | *p*-value |
| --- | --- | --- | --- | --- | --- | --- | --- | --- |
| Stomach/  Abdominal pain | No. of patients |  | 3 | 5 | 2^b^ | 3 | 0 | 0.238-^a^ |
|  | Days of AE | Mean ± SD | 1 | 1.2 ± 0.45 | 1.5 ± 0.71 | 1.3 ± 0.58 |  |  |
|  |  | Min-Max | 1 | 1 - 2 | 1 - 2 | 1 - 2 |  |  |
|  |  | Median | 1 | 1 | 1.5 | 1 |  |  |
|  | Grade of AE^k^ | 1 | 3 | 6 | 1 | 4 |  |  |
|  |  | 2 | 0 | 0 | 1 | 0 |  |  |
|  |  | 3 | 0 | 0 | 1 | 0 |  |  |
|  | Relation to treatment^l^ | probable | 2 | 4 | 2 | 0 |  |  |
|  |  | possible | 1 | 2 | 1 | 1 |  |  |
|  |  | remote | 0 | 0 | 0 | 1 |  |  |
|  |  | not related | 0 | 0 | 0 | 2 |  |  |
|  | Outcome^m^ | resolved spontaneously | 0 | 4 | 3 | 1 |  |  |
|  |  | resolved with treatment | 3 | 1 | 0 | 3 |  |  |
| Nausea | No. of patients | | 2 | 6 | 2^c^ | 1 | 1 | 0.116^a^ |
|  | Days of AE | Mean ± SD | 1 | 1 | 1.5 ± 0.71 | 1 | 1 |  |
|  |  | Min-Max | 1 | 1 | 1 - 2 | 1 | 1 |  |
|  |  | Median | 1 | 1 | 1.5 | 1 | 1 |  |
|  | Grade of AE^k^ | 1 | 2 | 4 | 2 | 1 | 1 |  |
|  |  | 2 | 0 | 2 | 1 | 0 | 0 |  |
|  | Relation to^l^ treatment | definite | 0 | 2 | 0 | 0 | 0 |  |
|  |  | probable | 1 | 3 | 1 | 0 | 1 |  |
|  |  | possible | 1 | 1 | 1 | 1 | 0 |  |
|  |  | not related | 0 | 0 | 1 | 0 | 0 |  |
|  | Outcome^m^ | resolved spontaneously | 0 | 6 | 2 | 1 | 1 |  |
|  |  | resolved with treatment | 2 | 0 | 1 | 0 | 0 |  |
| Dizziness | No. of patients | | 0 | 0 | 5^d^ | 1 | 0 | **0.001^a^** |
|  | Days of AE | Mean ± SD |  |  | 1.2 ± 0.45 | 1 |  |  |
|  |  | Min-Max |  |  | 1 - 2 | 1 |  |  |
|  |  | Median |  |  | 1 | 1 |  |  |
|  | Grade of AE^k^ | 1 |  |  | 6 | 1 |  |  |
|  | Relation to treatment^l^ | probable |  |  | 5 | 1 |  |  |
|  |  | remote |  |  | 1 | 0 |  |  |
|  | Outcome^m^ | resolved spontaneously |  |  | 6 | 1 |  |  |
| Vomiting | No. of patients | | 3 | 1 | 1 | 1 | 0 | 0.521^a^ |
|  | Days of AE |  | 1 | 1 | 1 | 1 |  |  |
|  | Grade of AE^k^ | 1 | 2 | 1 | 0 | 0 |  |  |
|  |  | 2 | 1 | 0 | 1 | 1 |  |  |
|  | Relation to treatment^l^ | probable | 1 | 0 | 0 | 1 |  |  |
|  |  | possible | 2 | 1 | 1 | 0 |  |  |
|  | Outcome^m^ | resolved spontaneously | 1 | 1 | 1 | 1 |  |  |
|  |  | resolved with treatment | 2 | 0 | 0 | 0 |  |  |
| Other | No. of patients | | 1^e^ | 3^f^ | 2^g^ | 2^h^ | 0 | n.d. |
|  | Days of AE |  | 1 | 1 | 1 | 1 |  |  |
|  | Grade of AE^k^ | 1 | 0 | 3 | 2 | 2 |  |  |
|  |  | 2 | 1 | 0 | 0 | 0 |  |  |
|  | Relation to treatment^l^ | probable | 1 | 0 | 0 | 0 |  |  |
|  |  | possible | 0 | 1 | 0 | 1 |  |  |
|  |  | not related | 0 | 2 | 1 | 1 |  |  |
|  | Outcome^m^ | resolved spontaneously | 0 | 0 | 1 | 1 |  |  |
|  |  | resolved with treatment | 1 | 2 | 1 | 0 |  |  |
|  |  | resolved with residual effect^i^ | 0 | 1 | 0 | 1 |  |  |

n.d. = not done

^a^ Fisher`s exact test

^b^The patient with two days abdominal pain had grade 1 on day 2 and grade 3 on day 8 (both resolving spontaneously)

^c^The patient with two days nausea had them on day 2 and day 13 (both grade 1)

^d^The patient with two days dizziness had them on two following days (day 2 and day 3, both days grade 1)

^e^Diarrhoea

^f^Headache, Itching, pain condition

^g^lethargy (fever), body pain

^h^General malaise, waist pain

^i^Both AEs with residual effect (waist pain, pain condition) were not related to treatment

^k^The Grading was always 1-3, to shorten this table only the grades which actually occurred are shown.

^l^The Relation to treatment could always be assessed as “definite”, “probable”, “possible”, “remote” and “not related”. To shorten this table only the relations which actually occurred are shown.

^m^The Outcome could always be assessed as “resolved spontaneously”, “resolved with treatment”, “resolved with residual effect”, “unchanged” and “death”. To shorten this table only the outcomes which actually occurred are shown.
