# Supplementary material for: Comparison of Doxycycline, Minocycline, Doxycycline plus Albendazole and Albendazole Alone in Their Efficacy against Onchocerciasis in a Randomized, Open-Label, Pilot Trial
Source: PLoS Negl Trop Dis. 2017 Jan 5;11(1):e0005156. doi: 10.1371/journal.pntd.0005156 (PMC5215804; doi:10.1371/journal.pntd.0005156)
Supplement: S3 Table — (DOCX) [file pntd.0005156.s003.docx]

**S3 table: ITT analysis – Effect of the study drugs on presence of *Wolbachia* in female worms: histology**

| Treatment Group | No. of Patients/ Nod ^a^ | No. of living female worms | | | |
| --- | --- | --- | --- | --- | --- |
|  |  | All | *Wolbachia* levels | | |
|  | 110/ 307 | 363 | many | few | none |
| DOX 4w (Standard) | 27/ 70 | 84 | 0 | 4 (4.8 %) | 80 (95.2 %) |
| DOX 3w + ALB 3d | 20/ 58 | 73 | 0 | 13 (17.8 %) | 60 (82.2 %) |
| MIN 3w | 21/ 58 | 73 | 0 | 19 (26.0 %) | 54 (74.0 %) |
| DOX 3w | 21/ 54 | 74 | 1 (1.4 %) | 27 (36.5 %) | 46 (62.2 %) |
| ALB 3d | 21/ 67 | 59 | 5 (8.5 %) | 32 (54.2 %) | 22 (37.3 %) |

^a^ Only evaluable patients/nodules are included.
