# Supplementary material for: Comparison of Doxycycline, Minocycline, Doxycycline plus Albendazole and Albendazole Alone in Their Efficacy against Onchocerciasis in a Randomized, Open-Label, Pilot Trial
Source: PLoS Negl Trop Dis. 2017 Jan 5;11(1):e0005156. doi: 10.1371/journal.pntd.0005156 (PMC5215804; doi:10.1371/journal.pntd.0005156)
Supplement: S4 Table — (DOCX) [file pntd.0005156.s004.docx]

**S4 table: ITT analysis – Effect of the study drugs on presence of *Wolbachia* in male worms: histology**

| Treatment Group | No. of Patients/ Nod ^a^ | No. of living male worms | | | |
| --- | --- | --- | --- | --- | --- |
|  |  | All^b^ | *Wolbachia* levels | | |
|  | 110/ 307 | 131 | many | few | none |
| DOX 4w (Standard) | 27/ 70 | 27 | 0 | 4 (14.8 %) | 23 (85.2 %) |
| DOX 3w + ALB 3d | 20/ 58 | 24 | 3 (12.5 %) | 5 (20.8 %) | 16 (66.7 %) |
| MIN 3w | 21/ 58 | 28 | 4 (14.3 %) | 6 (21.4 %) | 18 (64.3 %) |
| DOX 3w | 21/ 54 | 28 | 3 (10.7 %) | 14 (50.0 %) | 11 (39.3 %) |
| ALB 3d | 21/ 67 | 24 | 2 (8.3 %) | 12 (50.0 %) | 10 (41.7 %) |

^a^ Only evaluable patients/nodules are included.
