# Supplementary material for: Comparison of Doxycycline, Minocycline, Doxycycline plus Albendazole and Albendazole Alone in Their Efficacy against Onchocerciasis in a Randomized, Open-Label, Pilot Trial
Source: PLoS Negl Trop Dis. 2017 Jan 5;11(1):e0005156. doi: 10.1371/journal.pntd.0005156 (PMC5215804; doi:10.1371/journal.pntd.0005156)
Supplement: S5 Table — (DOCX) [file pntd.0005156.s005.docx]

**S5 table: ITT analysis – Effect of the study drugs on presence of *Wolbachia* in female worms: statistics^a,b^**

|  | DOX 3w + ALB 3d | MIN 3w | DOX 3w | ALB 3d |
| --- | --- | --- | --- | --- |
| DOX 4w | *p*=0.1473  OR 3.73 [0.63; 22.18] | *p*=0.0806  OR 5.12 [0.82; 31.95] | ***p*=0.017**  **OR 8.73 [1.47; 51.8]** | ***p*=0.0002**  **OR 29.16 [4.91; 173.2]** |
| DOX 3w + ALB 3d |  | *p*=0.5219  OR 1.41 [0.5; 3.99] | *p*=0.0726  OR 2.41 [0.92; 6.28] | ***p*<0.0001**  **OR 8.0 [3.14; 20.39]** |
| MIN 3w |  |  | *p*=0.2959  OR 1.71 [0.63; 4.65] | ***p*=0.0008**  **OR 5.70 [2.07; 15.72]** |
| DOX 3w |  |  |  | ***p*=0.0102**  **OR 3.34 [1.33; 8.39]** |

^a^ Alternating logistic regression (comparison of presence (many and few) vs. absence of *Wolbachia*)

^b^ Table shows the odds ratios (OR) for presence of *Wolbachia* comparing the treatment groups in the headline to the treatment groups in the left column.
