# Supplementary material for: Comparison of Doxycycline, Minocycline, Doxycycline plus Albendazole and Albendazole Alone in Their Efficacy against Onchocerciasis in a Randomized, Open-Label, Pilot Trial
Source: PLoS Negl Trop Dis. 2017 Jan 5;11(1):e0005156. doi: 10.1371/journal.pntd.0005156 (PMC5215804; doi:10.1371/journal.pntd.0005156)
Supplement: S6 Table — (DOCX) [file pntd.0005156.s006.docx]

**S6 table: ITT analysis – Effect of the study drugs on embryogenesis: histology**

| Treatment Group | No. of Patients/  Nod ^a^ | No.of living female worms | | | | | |
| --- | --- | --- | --- | --- | --- | --- | --- |
|  |  | All | Embryos | | | | Sperms in Uterus |
|  | 110/ 307 | 363 | not judgeable | oocytes only / uterus empty | normal | degenerated |  |
| DOX 4w (Standard) | 27/ 70 | 84 | 5 | 61 (77.2 %) | 6 (7.6 %) | 12 (15.2 %) | 10 (11.9 %) |
|  |  |  |  |  | (33.3 %)^b^ | (66.7 %)^b^ |  |
| DOX 3w + ALB 3d | 20/ 58 | 73 | 2 | 43 (60.6 %) | 10 (14.1 %) | 18 (25.4 %) | 14 (19.2 %) |
|  |  |  |  |  | (35.7 %)^b^ | 18 (64.3%)^b^ |  |
| MIN 3w | 21/ 58 | 73 | 7 | 45 (68.2 %) | 8 (17.9 %) | 13 (19.7 %) | 9 (12.3 %) |
|  |  |  |  |  | (38.1 %)^b^ | 13 (61.9 %)^b^ |  |
| DOX 3w | 21/ 54 | 74 | 2 | 49 (68.1 %) | 12 (16.7 %) | 11 (15.8 %) | 13 (17.6 %) |
|  |  |  |  |  | (52.2 %)^b^ | (47.8 %)^b^ |  |
| ALB 3d | 21/ 67 | 59 | 3 | 42 (75.0 %) | 10 (17.9 %) | 4 (7.1 %) | 9 (15.3 %) |
|  |  |  |  |  | (71.4 %)^b^ | (28.6 %)^b^ |  |

^a^ Only evaluable patients/nodules are included.

^b^ % of all female worms with embryogenesis within the respective group
