# Supplementary material for: Comparison of Doxycycline, Minocycline, Doxycycline plus Albendazole and Albendazole Alone in Their Efficacy against Onchocerciasis in a Randomized, Open-Label, Pilot Trial
Source: PLoS Negl Trop Dis. 2017 Jan 5;11(1):e0005156. doi: 10.1371/journal.pntd.0005156 (PMC5215804; doi:10.1371/journal.pntd.0005156)
Supplement: S7 Table — (DOCX) [file pntd.0005156.s007.docx]

**S7 table: ITT analysis – Effect of the study drugs on embryogenesis: statistics^a,b^**

|  |  | DOX 3w + ALB 3d | MIN 3w | DOX 3w | ALB 3d |
| --- | --- | --- | --- | --- | --- |
| DOX 4w |  | *p*=0.921  OR 1.05 [0.37;3.01] | *p*=0.6459  OR 1.36 [0.37;5.05] | *p*=0.0648  OR 1.94 [0.96;3.93] | ***p*=0.0303**  **OR 3.31 [1.12;9.78]** |
| DOX 3w + ALB 3d |  |  | *p*=0.843  OR 0.87 [0.23;3.32] | *p*=0.2351  OR 1.96 [0.64;5.99] | ***p*=0.0288**  **OR 4.48 [1.17;17.19]** |
| MIN 3w |  |  |  | *p*=0.4866  OR 1.64 [0.41;6.54] | *p*=0.1203  OR 3.38 [0.73;15.75] |
| DOX 3w |  |  |  |  | *p*=0.1978  OR 2.29 [0.65;8.12] |

^a^ alternating logistic regression, comparison of normal vs. degenerated embryogenesis

^b^ Table shows the odds ratio (OR) for embryogenesis comparing the treatment groups in the headline to the treatment groups in the left column.
