# Supplementary material for: Comparison of Doxycycline, Minocycline, Doxycycline plus Albendazole and Albendazole Alone in Their Efficacy against Onchocerciasis in a Randomized, Open-Label, Pilot Trial
Source: PLoS Negl Trop Dis. 2017 Jan 5;11(1):e0005156. doi: 10.1371/journal.pntd.0005156 (PMC5215804; doi:10.1371/journal.pntd.0005156)
Supplement: S8 Table — (DOCX) [file pntd.0005156.s008.docx]

**S8 table: ITT analysis – Effect of the study drugs on mf within the nodule: histology**

| Treatment Group | No. of Patients/  Nod^a^ | No. of Nodules^b^ | |
| --- | --- | --- | --- |
|  |  | All | with intact mf |
|  | 110/ 307 | 228 |  |
| DOX 4w | 27/ 70 | 56 | 0 |
| DOX 3w + ALB 3d | 20/ 58 | 49 | 4 (8.2 %) |
| MIN 3w | 21/ 58 | 43 | 2 (4.7 %) |
| DOX 3w | 21/ 54 | 39 | 3 (7.7 %) |
| ALB 3d | 21/ 67 | 41 | 6 (14.6 %) |

^a^ Only evaluable patients/nods are included

^b^ Only nodules with living female worms are included
