# Supplementary material for: Comparison of Doxycycline, Minocycline, Doxycycline plus Albendazole and Albendazole Alone in Their Efficacy against Onchocerciasis in a Randomized, Open-Label, Pilot Trial
Source: PLoS Negl Trop Dis. 2017 Jan 5;11(1):e0005156. doi: 10.1371/journal.pntd.0005156 (PMC5215804; doi:10.1371/journal.pntd.0005156)
Supplement: S9 Table — (DOCX) [file pntd.0005156.s009.docx]

**S9 table: ITT analysis – Effect of the study drugs on presence of *Wolbachia* in nodule sections: PCR^a^**

|  |  | DOX 4w | DOX 3w +  ALB 3d | MIN 3w | DOX 3w | ALB 3d |
| --- | --- | --- | --- | --- | --- | --- |
| FtsZ | N | 56 | 50 | 44 | 43 | 43 |
|  | Median | 518 | 835 | 3450 | 3250 | 8460 |
|  | 95% CI (Median) | 148;1194 | 416;2960 | 580;5670 | 1600;7570 | 4885;13600 |
|  | Percentiles 25^th^; 75^th^ | 93;2408 | 137;5730 | 255;8050 | 476;12700 | 2840;30100 |
|  | Min - Max | 0.06 - 56600 | 0.21 - 195000 | 0.03 - 99000 | 0 - 131000 | 0.27 - 130000 |
| Actin | N | 56 | 50 | 44 | 43 | 43 |
|  | Median | 1205 | 2005 | 864 | 1270 | 1250 |
|  | 95% CI (Median) | 935;2625 | 901;2970 | 448;2340 | 413;3030 | 565;2120 |
|  | Percentiles 25^th^; 75^th^ | 548;3250 | 635;5860 | 332;5325 | 265;6020 | 262;3380 |
|  | Min - Max | 4.30 - 27900 | 11.70 - 48600 | 9.18 - 405000 | 1.94 - 114000 | 37.30 - 45300 |
| FtsZ/Actin | N | 56 | 50 | 44 | 43 | 43 |
|  | Median | 0.26 | 0.50 | 1.10 | 1.45 | 8.20 |
|  | 95% CI (Median) | 0.09;0.86 | 0.39;0.75 | 0.43;2.49 | 1.12;4.03 | 2.12;22.82 |
|  | Percentiles 25^th^;75^th^ | 0.04;1.77 | 0.15;2.1 | 0.21;6.07 | 0.71;6.2 | 1.40;36.75 |
|  | Min - Max | 0 - 27.44 | 0 - 45.03 | 0 - 250.54 | 0 - 79.03 | 0 - 2832.24 |

^a^ Normally one PCR per nodule (from 3 nodules 2 PCRs were done and the mean was taken for further analyses (DOX 4w N = 2, DOX 3w N = 1), in 1 nodule PCR could not be performed (DOX 4w))
