# Supplementary material for: Comparison of Doxycycline, Minocycline, Doxycycline plus Albendazole and Albendazole Alone in Their Efficacy against Onchocerciasis in a Randomized, Open-Label, Pilot Trial
Source: PLoS Negl Trop Dis. 2017 Jan 5;11(1):e0005156. doi: 10.1371/journal.pntd.0005156 (PMC5215804; doi:10.1371/journal.pntd.0005156)
Supplement: S10 Table — (DOCX) [file pntd.0005156.s010.docx]

**S9 table: ITT analysis – Effect of the study drugs on presence of *Wolbachia* in nodule sections: statistics for FtsZ^a,b^**

|  |  | DOX 3w + ALB 3d | MIN 3w | DOX 3w | ALB 3d |
| --- | --- | --- | --- | --- | --- |
| DOX 4w |  | *p*=0.376 | *p*=0.1164 | ***p*=0.0477** | ***p*<0.0001** |
|  |  | OR 1.84 [0.48;7.11] | OR 3.1 [0.76;12.72] | **OR 4.23 [1.01;17.65]** | **OR 18.55 [5.7;60.39]** |
| DOX 3w + ALB 3d |  |  | *p*=0.4507 | *p*=0.2604 | ***p*<0.0001** |
|  |  |  | OR 1.69 [0.43;6.6] | OR 2.2 [0.56;8.67] | **OR 9.07 [3.0;27.41]** |
| MIN 3w |  |  |  | *p*=0.696 | ***p*=0.0045** |
|  |  |  |  | OR 1.33 [0.32;5.63] | **OR 5.65 [1.71;18.68]** |
| DOX 3w |  |  |  |  | ***p*=0.0167** |
|  |  |  |  |  | **OR 4.29 [1.3;14.16]** |

^a^ Alternating linear regression (after log_10_-transformation (all values +0.1 to circumvent zero values))

^b^ Table shows the odds ratio (OR) for embryogenesis comparing the treatment groups in the headline to the treatment groups in the left column.
