# Supplementary material for: Comparison of Doxycycline, Minocycline, Doxycycline plus Albendazole and Albendazole Alone in Their Efficacy against Onchocerciasis in a Randomized, Open-Label, Pilot Trial
Source: PLoS Negl Trop Dis. 2017 Jan 5;11(1):e0005156. doi: 10.1371/journal.pntd.0005156 (PMC5215804; doi:10.1371/journal.pntd.0005156)
Supplement: S11 Table — (DOCX) [file pntd.0005156.s011.docx]

**S11 table: ITT analysis – Effect of the study drugs on presence of *Wolbachia* in nodule sections: statistics for Actin^a,b^**

|  |  | DOX 3w + ALB 3d | MIN 3w | DOX 3w | ALB 3d |
| --- | --- | --- | --- | --- | --- |
| DOX 4w |  | *p*=0.5824 | *p*=0.7962 | *p*=0.5623 | *p*=0.8199 |
|  |  | OR 1.24 [0.57;2.69] | OR 1.12 [0.47;2.68] | OR 0.74 [0.26;2.08] | OR 0.91 [0.4;2.08] |
| DOX 3w + ALB 3d |  |  | *p*=0.8518 | *p*=0.3941 | *p*=0.4748 |
|  |  |  | OR 0.92 [0.38;2.24] | OR 0.63 [0.22;1.81] | OR 0.74 [0.32;1.7] |
| MIN 3w |  |  |  | *p*=0.591 | *p*=0.7244 |
|  |  |  |  | OR 0.74 [0.25;2.22] | OR 0.85 [0.34;2.13] |
| DOX 3w |  |  |  |  | *p*=0.6544 |
|  |  |  |  |  | OR 1.28 [0.43;3.78] |

^a^ Alternating linear regression (after log_10_-transformation (all values +0.1 to circumvent zero values))

^b^ Table shows the odds ratios (OR) for presence of *Wolbachia* comparing the treatment groups in the headline to the treatment groups in the left column.
