# Supplementary material for: Comparison of Doxycycline, Minocycline, Doxycycline plus Albendazole and Albendazole Alone in Their Efficacy against Onchocerciasis in a Randomized, Open-Label, Pilot Trial
Source: PLoS Negl Trop Dis. 2017 Jan 5;11(1):e0005156. doi: 10.1371/journal.pntd.0005156 (PMC5215804; doi:10.1371/journal.pntd.0005156)
Supplement: S12 Table — (DOCX) [file pntd.0005156.s012.docx]

**S12 table: ITT analysis – Effect of the study drugs on presence of *Wolbachia* in nodule sections: statistics for FtsZ/Actin^a,b^**

|  |  | DOX 3w + ALB 3d | MIN 3w | DOX 3w | ALB 3d |
| --- | --- | --- | --- | --- | --- |
| DOX 4w |  | *p*=0.8051 | ***p*=0.0397** | ***p*=0.002** | ***p*<0.0001** |
|  |  | OR 1.1 [0.52;2.35] | **OR 2.33 [1.04;5.22]** | **OR 3.27 [1.54;6.93]** | **OR 10.02 [4.17;24.11]** |
| DOX 3w + ALB 3d |  |  | *p*=0.1074 | *p*=0.0051 | ***p*<0.0001** |
|  |  |  | OR 1.92 [0.87;4.23 | OR 2.81 [1.36;5.79] | **OR 8.43 [3.58;19.84]** |
| MIN 3w |  |  |  | *p*=0.3565 | ***p*=0.0003** |
|  |  |  |  | OR 1.41 [0.68;2.95] | **OR 4.76 [2.03;11.18]** |
| DOX 3w |  |  |  |  | ***p*=0.0046** |
|  |  |  |  |  | **OR 3.32 [1.45;7.6]** |

^a^ Alternating linear regression (after log_10_-transformation (all values +0.1 to circumvent zero values))

^b^ Table shows the odds ratios (OR) comparing the treatment groups in the headline to the treatment groups in the left column.
