# Supplementary material for: Comparison of Doxycycline, Minocycline, Doxycycline plus Albendazole and Albendazole Alone in Their Efficacy against Onchocerciasis in a Randomized, Open-Label, Pilot Trial
Source: PLoS Negl Trop Dis. 2017 Jan 5;11(1):e0005156. doi: 10.1371/journal.pntd.0005156 (PMC5215804; doi:10.1371/journal.pntd.0005156)
Supplement: S13 Table — (DOCX) [file pntd.0005156.s013.docx]

**S13 table: ITT analysis – Microfilaridermia**

|  | Pre-treatment | | 6 months | |
| --- | --- | --- | --- | --- |
|  | Mf-positive | Mf-negative | Mf-positive | Mf-negative |
| DOX 4w | 19 (70.4%) | 8 (29.6%) | 18 (66.7%) | 9 (33.3%) |
| DOX 3w + ALB 3d | 14 (63.6%) | 8 (36.4%) | 15 (68.2%) | 7 (31.8%) |
| MIN 3w | 15 (64.2%) | 8 (34.8%) | 14 (60.9%) | 9 (39.1%) |
| DOX 3w | 14 (63.6%) | 8 (36.4%) | 13 (59.1%) | 9 (40.9%) |
| ALB 3d | 15 (68.2%) | 7 (31.8%) | 13 (59.1%) | 9 (40.9%) |
