# Supplementary material for: Comparison of Doxycycline, Minocycline, Doxycycline plus Albendazole and Albendazole Alone in Their Efficacy against Onchocerciasis in a Randomized, Open-Label, Pilot Trial
Source: PLoS Negl Trop Dis. 2017 Jan 5;11(1):e0005156. doi: 10.1371/journal.pntd.0005156 (PMC5215804; doi:10.1371/journal.pntd.0005156)
Supplement: S14 Table — (DOCX) [file pntd.0005156.s014.docx]

**S14 table: ITT analysis – Levels of Mf**

|  |  | Treatment | | | | |
| --- | --- | --- | --- | --- | --- | --- |
|  |  | DOX 4w | DOX 3w + ALB 3d | MIN 3w | DOX 3w | ALB 3d |
| Pre-treatment | N | 27 | 22 | 23 | 22 | 22 |
|  | Mean ± SD | 5.1 ± 7.1 | 5.9 ± 10.1 | 5.3 ± 13.1 | 5.4 ± 10.5 | 4.2 ± 9.3 |
|  | GM | 2.5 | 2.2 | 1.5 | 1.5 | 1.4 |
|  | Min - Max | 0 – 28.0 | 0 - 40.2 | 0 – 61.0 | 0 - 36.3 | 0 - 38.9 |
|  | Median | 2.6 | 1.3 | 0.3 | 0.3 | 0.5 |
|  | 95% CI (median) | 0.8;4.7 | 0;4.1 | 0;2.8 | 0;2.0 | 0.1;1.3 |
|  | Percentiles 25^th^;75^th^ | 0;5.3 | 0;4.8 | 0;2.8 | 0;2.3 | 0;3.8 |
| 6 months | N | 27 | 22 | 23 | 22 | 22 |
|  | Mean ± SD | 3.4 ± 7.0 | 4.4 ± 7.4 | 6.1 ± 13.6 | 8.7 ± 33.5 | 2.7 ± 6.6 |
|  | GM | 1.4 | 1.6 | 1.5 | 1,1 | 0,9 |
|  | Min - Max | 0 - 33.4 | 0 - 21.1 | 0 – 48.0 | 0 - 157.8 | 0 – 29.0 |
|  | Median | 0.6 | 0.7 | 0.3 | 0.4 | 0.2 |
|  | 95% CI (median) | 0;2.6 | 0;1.9 | 0;3 | 0;1.1 | 0;0.9 |
|  | Percentiles 25^th^;75^th^ | 0;4.2 | 0;2.5 | 0;4.6 | 0;1.2 | 0;1.4 |
|  | *p*-value^b^ | ***p*=0.028** | *p*=0.234 | *p*=0.711 | *p*=0.438 | ***p*=0.025** |

SD = standard deviation, GM = geometric mean

^a^ The geometric mean (GM) was calculated by adding 1 to all values and after the calculation 1 was again subtracted from the result

^b^ Wilcoxon-signed-rank-test

^c^ No difference between all 5 treatment groups pre-treatment (p=0.722, Kruskal-Wallis-test)

^d^ No difference between all 5 treatment groups at 6 months (p=0.862, Kruskal-Wallis-test)
